# A New Approach for CRISPR/Cas9 Editing and Selection of Pathogen-Resistant Plant Cells of Wine Grape cv. ‘Merlot’

**DOI:** 10.3390/ijms251810011

**Published:** 2024-09-17

**Authors:** Anastasia Fizikova, Zhanneta Tukhuzheva, Lada Zhokhova, Varvara Tvorogova, Ludmila Lutova

**Affiliations:** 1 Plant Biology and Biotechnology Department, Sirius University of Science and Technology, Olympic Avenue 1, 354340 Sochi, Russiala.lutova@gmail.com (L.L.); 2 Department of Genetics and Biotechnology, Saint-Petersburg State University, Universitetskaya Emb 7/9, 199034 Saint-Petersburg, Russia

**Keywords:** CRISPR/Cas9, plant biotechnology, grape editing, plant resistance, mildew

## Abstract

Grape is one of the most economically significant berry crops. Owing to the biological characteristics of grapes, such as the long juvenile period (5–8 years), high degree of genome heterozygosity, and the frequent occurrence of inbreeding depression, homozygosity during crossbreeding leads to loss of varietal characteristics and viability. CRISPR/Cas editing has become the tool of choice for improving elite technical grape varieties. This study provides the first evidence of a decrease in the total fraction of phenolic compounds and an increase in the concentration of peroxide compounds in grape callus cells upon the addition of chitosan to the culture medium. These previously unreported metabolic features of the grape response to chitosan have been described and used for the first time to increase the probability of selecting plant cells with *MLO7* knockout characterised by an oxidative burst in response to the presence of a pathogen modulated by chitosan in the high-metabolite black grape variety ‘Merlot’. This was achieved by using a CRISPR/Cas9 editing vector construction with the peroxide sensor HyPer as a reporter. This research represents the first CRISPR/Cas9 editing of ‘Merlot’, one of the most economically important elite technical grape varieties.

## 1. Introduction

Grape is one of the oldest cultivated plants, estimated to have been grown by man for six to eight thousand years. *Vitis vinifera* L. is the only species of the genus native to Eurasia, and is thought to have originated around 65 million years ago, laying the foundations for wine-making. The first documented variety, “Gue Blanc”, dates back to 1283. Since ancient times, viticulture has been considered a primary branch of agriculture due to the nutritional value and uniqueness of the vine [1]. Grape is a valuable source of B vitamins, antioxidants, bioflavonoids, and melatonin. It is not only a cultural heritage of humanity, but the product also has a unique composition, which includes valuable metabolites, such as caffeic, gallic, and coumaric acids, trans-resveratrol, flavonoids (rutin, quercetin, myricetin, and catechins), as well as indoles, such as melatonin, serotonin, and tryptamine [2,3]. The unique composition of wine determines its anti-inflammatory, protective properties, reducing the likelihood of developing many neurodegenerative and cardiovascular diseases. Wine has anti-inflammatory and hypolipidaemic effects [4,5], promotes proper circadian rhythms, and contributes to longevity [6,7]. Only 12% of grapes are consumed in their raw form, with the majority being used for wine production (about 60% of varieties) [1,8]. Through gradual domestication, selection, and breeding, humans have developed about 12,000 grape varieties, 11 of which account for more than one-third of the world’s vineyards due to their unique bouquet, acidity, and tannin composition. The bases of technical viticulture and mature winemaking throughout the world are the so-called “Nobel” varieties: ‘Cabernet Sauvignon’, ‘Merlot’, ‘Chardonnay’, ‘Syrah’, ‘Sauvignon Blanc’, ‘Pinot Noir’, ‘Riesling’, ‘Chasselas’, ‘Grenache’, ‘Monastrell’, and ‘Ugni Blanc’ [9]. The susceptibility of these unique technical grape varieties to fungal pathogens necessitates annual preventive treatments with fungicides, significantly impacting the ecological health of wine-growing regions and fuelling the emergence of fungicide-resistant pathogens [10,11]. Attempts to obtain pathogen-resistant technical grape varieties using traditional methods of hybridisation, selection, and marker-assisted selection (MAS) are extremely laborious, take a long time due to the biological characteristics of the culture, and often lead to the loss of unique varietal characteristics and winemaking prospects in the selected progeny. Partly because of the negative experience in attempts to select resistant technical grape varieties that could compete in terms of wine bouquet complexity with the varieties on the “Nobel” list, the world of mature wine-making has become extremely conservative, cautious, and often negatively disposed to the use of hybrids on an industrial scale, as this is associated with potential risks [12,13]. Therefore, finding new approaches to obtain grape varieties resistant to fungal pathogens is an important task in modern plant biotechnology [14].

### 1.1. Molecular Mechanisms of Plant Defense against Pathogens

Plants have a two-stage defence system against pathogens: PTI (pathogen-associated molecular pattern-triggered immunity) and ETI (effector-triggered immunity) [15]. PTI is activated when plant receptors (PRRs) on the cell surface recognise pathogen-associated molecules (PAMPs), such as those from *E. necator*, triggering an initial immune response [16]. ETI, on the other hand, is triggered by pathogen virulence factors that are recognised directly or indirectly recognised by intracellular receptors in the plant, often associated with resistance-conferring R genes [17]. Both PTI and ETI activate signalling pathways that lead to various defence responses including the activation of enzymes such as NADPH oxidase and cell wall peroxidases, leading to an oxidative burst that generates reactive oxygen species (ROS) [18]. This oxidative burst can trigger the hypersensitive response (HR), a localised programmed cell death that effectively isolates the infected area and prevents the pathogen from spreading to adjacent tissues [19]. HR is not simply a destructive response; it also serves as a key signal that activates various defence pathways and phytohormone synthesis, further enhancing plant resistance and preventing disease development [20].

Pathogens release elicitors, including PAMPs and effectors, which are perceived by plants as danger signals. These elicitors are recognised by cell-surface PRRs and intracellular NLRs (nucleotide-binding receptors with leucine-rich repeats), which activate the PTI and ETI pathways, respectively. An example of a fungal PAMP is chitin, which is found in the cell walls of fungi [20].

The recognition of elicitors triggers signalling cascades within the plant, leading to a range of defence responses. These can include stomatal closure [21,22], limitation of nutrient translocation, production of antimicrobial compounds [23,24], ROS production, and programmed cell death (PCD) at the site of infection [25]. Plant responses to pathogens also involve phytohormones, such as salicylic acid (SA), jasmonic acid (JA), and ethylene. SA primarily regulates defence against biotrophic pathogens, whereas JA and ethylene are more involved in responses to necrotrophic pathogens [26]. The activation of the SA pathway leads to systemic acquired resistance (SAR) by inducing PR genes that encode antimicrobial proteins, thereby increasing resistance to a wider range of pathogens [27,28].

Grapes, like many plants, have complex defence mechanisms against pathogens. One strategy involves resistance genes, such as *REN* and *RUN*, which trigger a hypersensitive response (HR) at the site of infection, effectively isolating the pathogen [29]. This HR “burst” involves rapid cell death, preventing the spread of infection. While resistance genes are valuable for disease control, an alternative approach uses susceptibility S genes. Loss of function in these genes can lead to recessive resistance, although this strategy can have unintended consequences. The suppression of S-genes can result in reduced yield, altered morphology and metabolism, disrupted interactions with beneficial microorganisms, and changes in stress tolerance [30,31].

Notable among the S genes are the *MLO* (powdery mildew locus O) genes, which are highly conserved across different plant species. Loss of function in these genes confers recessive resistance to powdery mildew in barley, *Arabidopsis thaliana*, pea, tomato, wheat, and pepper [30,32,33,34,35,36]. Seventeen *MLO* genes have been identified in grape, and the suppression of the *MLO6*, *MLO7*, and *MLO11* genes has been shown to increase resistance to *E. necator* [37,38]. Three grape *MLO* genes (*VvMLO7*, *VvMLO11*, and *VvMLO13*) are activated early in the infection process, suggesting a role in regulating defence pathways [39,40]. MLO proteins are considered to be negative regulators of vesicle-associated, actin-dependent defence pathways at the site of powdery mildew penetration [39]. These vesicles play a crucial role in pathogen penetration, allowing the formation of papillae on the cell wall, which are associated with *MLO* resistance [41]. Studies have shown that powdery mildew-induced mesophyll cell death, cell wall apposition, and H_2_O_2_ production are associated with *mlo*-mediated resistance. *VvMLO3*-edited grapevine plants exhibit infection-induced cell death, H_2_O_2_ accumulation, and cell wall apposition (CWA) [39], suggesting that *MLO* genes play an important role in regulating the plant’s defence response to powdery mildew infection.

Using tools such as CRISPR/Cas editing systems, we can potentially manipulate plant defence responses by pre-emptively “informing” the plant of an impending pathogen encounter. This could involve precisely modifying the plant’s metabolic pathways to enhance its innate resistance, or fine-tuning its response mechanisms to minimise the detrimental effects of infection.

### 1.2. CRISPR/Cas Editing of Grapes

Due to the inherent biological characteristics of grapes, including the extended juvenile period of 5–8 years, high degree of genome heterozygosity, and the common occurrence of inbreeding depression, leading to a loss of varietal traits and viability when crossed [42,43,44], CRISPR/Cas9 genome editing has emerged as the tool of choice for improving elite technical grape cultivars [45,46]. CRISPR/Cas9 allows the preservation of a plant’s unique heterozygous state without affecting the rest of the genome when generating non-transgenic modified plants with mutations in specific loci. This mutagenesis-focused technology provides opportunities for epigenome editing to fine-tune, suppress, or activate gene function [47,48].

Since 2016, the grape genome has been successfully edited using CRISPR/Cas [49,50,51,52,53,54,55,56,57,58,59,60,61,62,63,64,65]. Comparing the editing efficiency across studies is challenging due to the wide range of different methods used by researchers. These variations include: (1) transfection methods: PEG [50,53] and lipofectamine [64]-mediated protoplast transfection and *Agrobacterium*-mediated vector transfection [49,51,52,54,55,56,57,58,59,60,61,62,63,65]; (2) promoters for gRNA and Cas9 expression: AtU6, VvU6.1, VvU6.2, and OsU3 for gRNA and CaMV35S, VvUBQ2, and VvUBI2 for Cas9; (3) nucleases: SpCas9, LbCas12a, zCas9i, hCas9, and PE; (4) selection approaches: antibiotic resistance (KnR [51,52,55,58,59,63], HygR [49,56,65]) or fluorescence (GFP [53,57,60,62,64,65] or RFP [63]); (5) target genes for knockout: *PDS1*, *TMT1*, *TMT2*, *DFR1*, *DXS1*, *IdnDH*, *MLO7*, *MLO3*, *MLO4*, *MLO6*, *CCD7*, *PR4b*, *TAS4b*, *MYBA7*, *AIR12*, *SWEET4*, *LIN2*, *DEL1*, *DMR6*, *WRKY52*, and *MADS45* [49,50,51,52,53,54,55,56,57,58,59,60,61,62,63,64,65], resulting in varying gRNA efficiencies; (6) amount of gRNA per target; (7) grape varieties; and (8) sequencing and counting of editing efficiency: NGS [50,61,62,64] or Sanger sequencing of selected calli (including the approach of hydrolysing sequences with restriction endonucleases, which artificially increases the frequency of indel mutations) [51,59,65]), selected plantlets [58], or plants [55,57,60,62,64].

Despite these differences, it is clear that a limited number of grape varieties have been genetically edited to date. These include the rootstocks 101-14 (*Riparia* × *Rupestris* group) [55] and 41B (*Chasselas* × *Berlandieri*) [57,59,62], the table grape varieties ‘Thompson Seedless’ [52,54,56,58,60], ‘Sugraone’ [61], ‘Neo Muscat’ [51], ‘Red Globe’ [54], ‘Crimson seedless’ [61], and ‘Scarlet Royal’ [65], and the wine varieties ‘Chardonnay’ [49,50,53,63] and ‘Nebbiolo’ [64]. It is noteworthy that only ‘Chardonnay’, a white wine variety, has been edited from the list of the most planted economically important wine varieties in published works. CRISPR/Cas-mediated genome editing studies in grapes have explored different approaches to confer resistance to major grape pathogens: knockouts of the *DMR6* and *PR4b* genes [56,61] have been shown to confer resistance to downy mildew; targeting of the *WRKY52* gene [52] has been found to improve resistance to grey rot; and editing of the *MLO3*, *MLO4* [58], *MLO6* [61], and *MLO7* [50] genes has been reported to improve resistance to powdery mildew.

These studies demonstrate the potential of CRISPR/Cas-based genome editing to create grapevine varieties with increased resistance to major fungal pathogens. However, despite this potential, successful CRISPR/Cas editing efforts have yet to be reported for other major, economically important “Nobel” wine grape varieties beyond ‘Chardonnay’. These include ‘Cabernet Sauvignon’, ‘Syrah’, ‘Sauvignon Blanc’, ‘Pinot Noir’, ‘Riesling’, ‘Chasselas’, ‘Grenache’, ‘Monastrell’, ‘Ugni Blanc’, and ‘Merlot’.

## 2. Results

**Figure 1:**
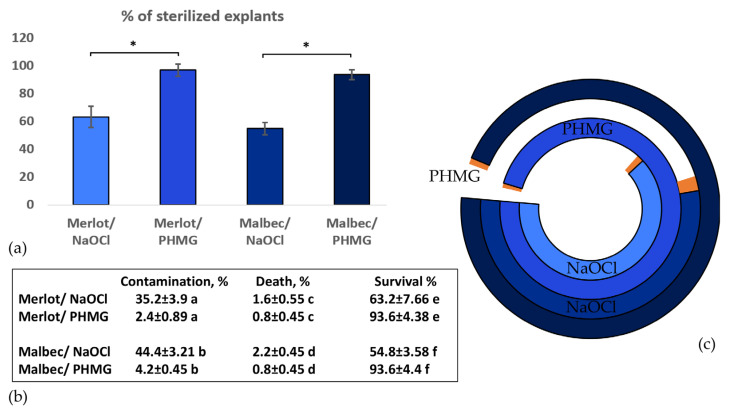
Comparison of the effectiveness of grapevine leaf bud sterilisation using sodium hypochlorite (NaOCl) and polyhexamethylene guanidine hydrochloride (PHMG); the statistical significance of the observed differences was assessed using a two-tailed Mann–Whitney U test: (**a**) Histogram comparing the percentages of sterilised explants on day 4 of incubation: the analysis revealed a statistically significant difference, with: * ‘Merlot’ PHMG decontamination having a higher percentage of sterilised explants compared with ‘Merlot’ NaOCl decontamination approach (z = −2.69, *p* = 0.008); * ‘Malbec’ PHMG decontamination having a higher percentage of sterilised explants compared with ‘Malbec’ NaOCl decontamination approach (z = −2.74, *p* = 0.008); (**b**) Table comparing the two sterilisation methods based on percentages of contaminated, dead, and surviving explants after 3 weeks of incubation: the relative values marked with the same letter were compared with each other and showed statistically significant differences: (a) the difference was statistically significant at *p* = 0.006 (z = −2.73); (b) the difference was statistically significant at *p* = 0.007 (z = −2.69); (c) the difference was statistically significant at *p* = 0.042 (z = −2.03); (d) the difference was statistically significant at *p* = 0.005 (z = −2.79); (e) the difference was statistically significant at *p* = 0.008 (z = −2.69); (f) the difference was statistically significant at *p* = 0.008 (z = −2.74); (**c**) Circular chart showing the percentages of surviving (blue) and dead (orange) explants: The exact percentages can be seen in (**b**) of Figure 1: parts of the rings corresponding to contaminated samples are not coloured.

The main objective of this work was to develop an approach for CRISPR/Cas editing of high-metabolite black varieties of technical grapes from the “Nobel” list. To this end, at the first stage of our research, axillary and apical buds, collected from vineyards of the ‘Malbec’ and ‘Merlot’ varieties were sterilised and introduced into in vitro cultures. The effectiveness of two sterilisation methods, using NaOCl and PHMG, was compared (Figure 1). As shown in Figure 1, the use of PHMG (polyhexamethylene guanidine hydrochloride) represents a new and significantly more effective decontamination method for grapevine explants compared with the conventional NaOCl (sodium hypochlorite) treatment. The PHMG protocol resulted in a higher percentage of sterilised explants with lower mortality rates.

### 2.1. Micropropagation

In order to determine the most suitable conditions for the microclonal propagation of black technical cultivars, we carried out a visual experiment to assess the phenotype of cuttings from both white and black technical cultivars when transferred to different media used for microclonal propagation. We used 2–3 cuttings from each plant (as technical replicates) and included 10 plants of each variety to increase the likelihood of identifying any potential patterns. We selected media from the commercial microclonal propagation method developed by PlantCellTechnology owing to their versatility. These media included 1M (MS 0.5 mg/L BAP), 2M (MS 1 mg/l BAP, 0.1 mg/L IBA), 3M (MS 4 mg/L IAA), and a medium that has performed well in the propagating local table hybrid varieties and is protected by a patent RU2041609, 4M (MS 0.2 mg/L IBA).

**Figure 2:**
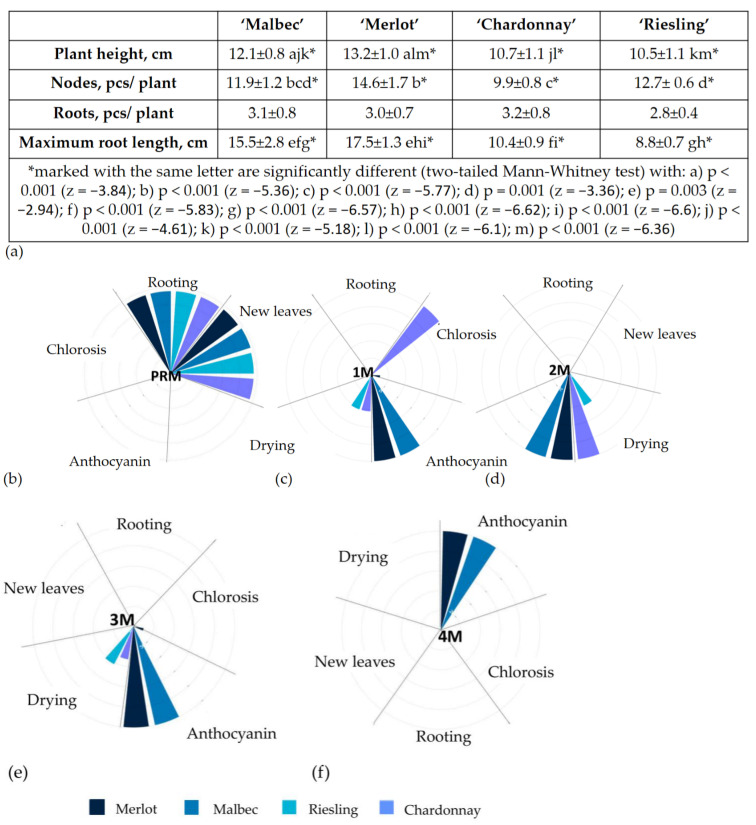
Comparison of the phenotypes of cv. ‘Merlot’, ‘Chardonnay’, ‘Malbec’, and ‘Riesling’ cuttings on PRM and 1M–4M media: (**a**) Comparison of the growth parameters of vine cuttings of the varieties ‘Merlot’, ‘Chardonnay’, ‘Malbec’, and ‘Riesling’ on PRM medium over a period of one month, without any subculturing, showed significant differences in shoot height, number of nodes, and root lengths across the different varieties. However, there were no statistically significant differences observed in the number of roots formed by the ‘Merlot’, ‘Chardonnay’, ‘Malbec’, and ‘Riesling’ cuttings (Figure 2a). (**b**) Polar area plot illustrating the range of phenotypes observed when cultivating grapevine cuttings of the ‘Merlot’, ‘Chardonnay’, ‘Malbec’, and ‘Riesling’ varieties on PRM medium after three weeks of cultivation, showing a statistically significant preference for growth on PRM medium (Fisher test, F = 625, *p* < 0.0001). The polar area plots (**c**–**f**) show the phenotypic responses of ‘Merlot’, ‘Chardonnay’, ‘Malbec’, and ‘Riesling’ grapevine cuttings grown on 1M–4M media after three weeks. A statistically significant difference (F = 841, *p* < 0.001) was observed in the accumulation of anthocyanins in black grape varieties (‘Merlot’ and ‘Malbec’) compared with white grape varieties (‘Chardonnay’ and ‘Riesling’) on 1M–4M media. This difference was also observed in the development of chlorosis in ‘Chardonnay’ cuttings on 1M media (F = 841, *p* < 0.001). Furthermore, a significant difference (F = 169, *p* = 0; F = 100, *p* = 0 for 2M and 3M media, respectively) was observed in the drying of buds in white grape varieties compared with black grape varieties on 1M, 2M, and 3M media.

In addition, we have included a medium that we randomly decided to try, which currently seems to be optimal for working with metabolic grape varieties: PRM (WPM 0.2 mg/L NAA, 0.2 mg/L 6-BAP). After three weeks of cultivation, the differences observed were already striking (Figure 2): some cuttings accumulated anthocyanin (black grape ‘Merlot’ and ‘Malbec’ on media 1M–4M) (Figure 2c–f), chlorosis appeared on some leaves (white grape ‘Chardonnay’ on 1M and white grape ‘Riesling’ on 3M (Figure 2c,e)), and the upper part of the shoots of ‘Chardonnay’ and ‘Riesling’ dried out on 1M–3M (Figure 2c,e). Callus formation occurred only on the second medium, specifically on the part of the shoot immersed in the medium. No new leaves or roots were observed on the 1M–4M media (Figure 2c–f).

Prolonged cultivation resulted in leaf drop and death of the cuttings, except for those cultivated on the PRM medium. The new PRM medium for grape cultivation (WPM 0.2 mg/L NAA, and 0.2 mg/L 6-BAP) showed significantly higher success in rooting and leaf formation compared with the other tested media (1M, 2M, 3M, and 4M). After 3 weeks of cultivation on PRM, most of the cuttings showed both rooting and new leaf growth, a statistically significant difference (*p* < 0.001) from the other media, where no such development occurred. Cuttings of ‘Merlot’, ‘Chardonnay’, ‘Malbec’, and ‘Riesling’ showed root and leaf growth after 2–3 weeks on PRM, while no such growth was observed on other media after 3 weeks or longer (Figure 2a). Cuttings on media 1M–4M showed gradual wilting. ‘Merlot’ and ‘Malbec’ cuttings accumulated anthocyanin on media 1M–4M (Figure 2a,c–f), but did not form roots or new leaves.

The observed phenotypic differences showed that cuttings of ‘Merlot’, ‘Malbec’, ‘Chardonnay’, and ‘Riesling’ grown on the tested media showed differences after 3 weeks of cultivation. Explants grown on these media accumulated anthocyanins, and showed bud necrosis and chlorosis, indicating unfavourable changes in plant physiology and metabolism due to the stress experienced [66,67]. This highlights the importance of understanding the metabolic differences of a wide range of grape cultivars in order to have a means to reduce stress in explants, both when working with in vitro cultures and when developing methodologies for the transfection and regeneration of different grape cultivars according to their unique metabolic features and characteristics.

### 2.2. Evaluation of Phenolic Metabolite and Peroxide Content in ‘Merlot’ Grapevine Cells upon Induction of Plant Immune Response (PTI) by Chitin

**Figure 3:**
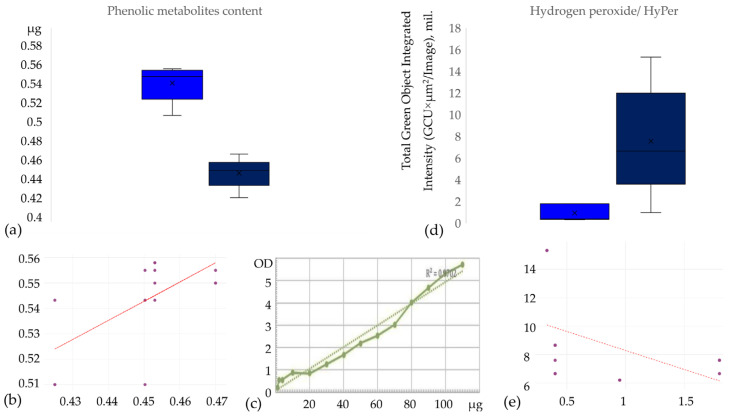
Induction of plant immune response by chitosan in ‘Merlot’ grapevine suspension callus: (**a**) box plot showing total phenolic metabolite content in ‘Merlot’ grapevine suspension callus with (navy blue) and without (cornflower blue) chitosan treatment: *y*-axis: total phenolic metabolite content (µg per probe), *x*-axis: treatment (control, chitosan), significant difference observed between groups (*t*-test, *p* < 0.001); (**b**) scatter plot showing correlation between total phenolic metabolite content in ‘Merlot’ grapevine suspension callus in two samples with and without chitosan treatment (Rs = 0.6063 *p* = 0.02 (98% statistical significance level) df = 0; R^2^ = 0.39); (**c**) calibration curve for the determination of the total phenolic metabolite content: *y*-axis: absorbance at 700 nm; *x*-axis: total phenolic metabolite concentration (µg per probe), R^2^ = 0.9702; (**d**) box plot showing the average intensity values of the number of fluorescent objects (sensor for peroxide compounds (HyPer)) in ‘Merlot’ grape callus cells with (navy blue) and without (cornflower blue) chitosan addition. Measurements were taken at 9 points using a spiral imaging strategy with the Incucyte live cell analysis system (500 nm). A significant difference was observed between the groups (U-test: U = −2.61, *p* < 0.001). (**e**) Scatter plot shows the correlation between the content of fluorescent objects in ‘Merlot’ samples with and without chitosan treatment (Rs = −0.5991 *p* = 0.05 (95% statistical significance level) df = 0; R^2^ = 0.2).

In order to understand what metabolic changes occur during the activation of the immune response to a pathogen, we evaluated the correlation between the total fraction of phenolic metabolites and peroxide compounds in suspension callus cells when cultured with chitosan. To characterise the suspension callus in terms of phenolic metabolite and peroxide contents, we induced loose callus on the medium (MS with 2.4 D 9 mg/L and 4.5 mg/L 6-BAP), and cultured it on MS (3 mg/L BAP and 1 mg/L NAA). Callus was transfected with a vector carrying the HyPer sensor as a reporter. After the decontamination of the culture from *Agrobacterium* (three weeks after inoculation), callus suspensions were divided into equal parts. Chitosan was added to half of each sample to simulate infection by a fungal pathogen and induce an immune response in the cells. Callus suspensions were incubated in liquid media containing either chitosan or a control medium without chitosan under constant rotation in a rotary shaker (100 rpm) for three days. The concentration of the total phenolic metabolite fraction of callus cells and HyPer fluorescence, which correlates with intracellular hydrogen peroxide levels, were then determined [68]. All samples were measured in triplicate and with two biological replicates (Figure 3).

As shown in Figure 3, there is a statistically significant decrease in the content of phenolic metabolites and an increase in the content of peroxide compounds in callus cells upon the addition of chitosan.

### 2.3. CRISPR/Cas9 Genome Editing of ‘Merlot’ Callus and Proembryogenic Mass

**Figure 4:**
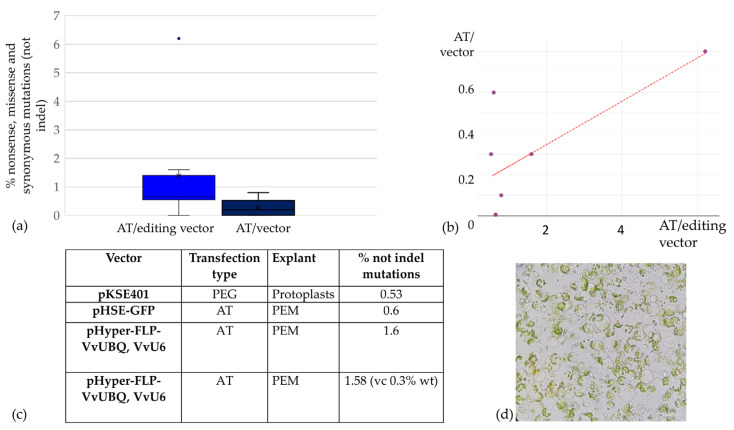
Increased levels of nonsense, missense, and synonymous mutations following agrobacterial transfections with editing vectors: (**a**) Box plot representing the percentage of nonsense, missense, and synonymous mutations occurring after *Agrobacterium*-mediated transfections (AT) with editing vectors. Cells that underwent *Agrobacterium*-mediated transfection without gRNA were used as a control. The significance of the observed differences was analysed using the Mann–Whitney test: (Z = −2.37, *p* < 0.05). (**b**) Scatter plot showing the correlation between control and treated experimental groups. (**c**) Table showing mutation percentages from primary experiments, indicating vector types, explants used, and transfection methods; mutation frequencies in these experiments were assessed using Mi-Seq NGS; (**d**) Microscopy of one of the explants used in this study: grapevine protoplasts of the variety ‘Merlot’; bright field, scale bar 50 μm.

The next stage of this work was dedicated to optimising the genome editing of black technical grape varieties, taking advantage of the demonstrated reduction in the amount of phenolic metabolites in explants upon the addition of chitosan, and aimed to explore the potential of CRISPR/Cas9 genome editing to improve the metabolic traits of elite black wine grapes, specifically ‘Merlot’ and ‘Malbec’. We used a conventional vector-based system to deliver CRISPR/Cas9 components into plant cells, a widely used vector that has been successfully used for genome editing in various plant species over the past decade. This system is based on the pKSE401 and pHSE401 vectors [69]. Our efforts included several rounds of optimisation for the construction of the editing vectors. These modifications included (1) reporter gene integration: replacement of the selective marker with the EGFP reporter gene, allowing the removal of antibiotic selection pressure and increasing regeneration efficiency by selecting transfected explants based on fluorescence; and (2) grapevine promoter substitution: replacement of promoters for shRNA and Cas9 expression with grapevine-specific *VvU6* and *VvUBQ2* promoters [59]. Inducible excision system: introduction of a sequence encoding a flippase under a heat-inducible promoter, together with recognition sites for the flippase. This modification allows for the inducible excision of editing constructs, paving the way for the potential generation of non-transgenic edited plants in the future [70]. A variety of explant types have been tested, including protoplasts [50,53,64], callus suspension, and cultivation on sterile filters. The protocol for the enrichment of ‘Merlot’ and ‘Malbec’ calli with pro-embryogenic cells was optimised, and both PEG-mediated and *Agrobacterium*-mediated transformations were carried out. Various mutation detection methods and strategies were also used, including Sanger sequencing, NGS, and T7EI. However, despite these efforts, we were unlucky to find any deletions or insertions (indels) in our target sequences (Figure 4).

**Figure 5:**
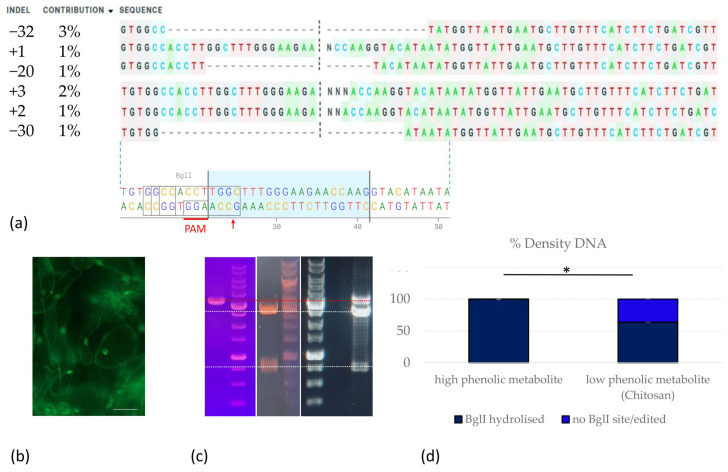
Evaluation of the CRISPR/Cas9 gene-editing efficiency of *MLO7* in the grapevine variety ‘Merlot’: (**a**) Aligned nucleotide sequences of the *MLO7* gene: the region where the Cas9 nuclease is most likely to induce a break in the sequence is marked by a black dashed line. Blue dashed lines indicate correspondence with the unedited *MLO7* sequence, the guide RNA region is highlighted in blue, the PAM site is marked in red, and the localisation of the *BglI* restriction endonuclease recognition site used for *MLO7* PCR product screening is indicated; (**b**) Fluorescence microscopy of callus cells transfected with the editing construct expressing the hydrogen peroxide sensor HyPer, scale bar 20 μm; (**c**) Electropherograms of *MLO7* PCR products and restriction fragments after *BglI* hydrolysis: from left to right, samples: 1. *MLO7* non treated; 2. 1 kb DNA ladder (Evrogen); 3. *MLO7*/*BglI* (after AT with an editing vector); 4. 1000 bp DNA ladder (Thermo Fisher, Waltham, MA, USA); 5. 1 kb DNA ladder (Evrogen); 6. *MLO7*/*BglI* (after AT with an editing vector with chitosan treatment of explants); (**d**) Densitogram analysis of electrophoregrams to quantify the *BglI*-hydrolysed *MLO7* sequence (36.3% ± 0.006) (GelAnalyzer): * the difference was statistically significant at *p* < 0.05 (Z = –2.31). The ‘low phenolic metabolite’ (new transfection and selection approach with chitosan supplementary) had a higher percentage of BglI site loss (edited) *MLO7* sequence (or KO *MLO7*) compared with the standard CRISPR/Cas9 editing and *Agrobacterium*-mediated transfection approach.

Next, we chose to use liquid culture supplemented with chitosan and PVP during the preparation of ‘Merlot’ grape explants for transfection with the HyPer-containing editing vector. The aim of this approach was to reduce the metabolic burden on the explant cells by suppressing the production of phenolic metabolites. The selection of transfected cells was to be guided by HyPer fluorescence, based on the previously described increase in oxidative stress and the production of peroxide compounds in plant cells with knockouts of *MLO* genes. This oxidative response at the site of fungal infection is thought to underlie the frequently observed development of fungal pathogen resistance in *MLO* mutant plants [10]. Therefore, at all stages after transfection up to the genotyping, the explants were washed with media containing chitosan and PVP: every three days for the first two weeks after co-cultivation, replacing the media with cefotaxime and charcoal (PRM), and then every week. About two months later, after observing the growth of the callus with HyPer fluorescence, we performed cell genotyping (Figure 5).

When aligning and analysing the amplified sequences, the editing efficiency in the heterogeneous pool of PCR products was 9%. We did not artificially increase the screening resolution by purifying a non-hydrolysed edited pool of amplicons, as has been performed in other studies. In addition, while using Sanger/ICE screening, T7E1 mismatch detection assay, and Mi-Seq sequencing at the previous stages (without chitosan, PVP pre-treatment of explants), we did not detect any sequences with deletions or insertions in the amplicon libraries (five experiments on ‘Merlot’); treatment of explants with chitosan and PVP, cultivation on charcoal-containing media, and selection based on HyPer fluorescence significantly improved the probability of editing and allowed the selection of cells with deletions of −32 bp (3%), −30 bp (1%), and −20 bp (1%), as well as insertions of +1 bp, +2 bp, and +3 bp. The frequency of the introduced knockouts increased to the point where they could even be detected by Sanger sequencing and ICE detection of the *MLO7* target amplified on a heterogeneous pool of transfected HyPer^+^ callus cells.

## 3. Discussion

Grapevines are highly metabolic plants, and the metabolic differences between varieties are particularly contrasting and evident in technical grape varieties. This complicates the process of comparing the results obtained due to the differences in the metabolic composition of different varieties and different approaches to assessing the efficiency of transformation and regeneration. The unique characteristics of woody plants, such as their long life cycle, slow growth and development, and large number of secondary metabolites are, on the one hand, qualities that we value greatly in culture, but on the other hand, are qualities that make it very difficult to work with and dictate the need for careful planning of upcoming experiments. If a poor choice is made and an inappropriate technique is used, a significant amount of irreplaceable resources, such as time, can be lost.

Despite the fact that work on grapes editing began in 2016, only some varieties of this complex crop have been successfully edited: only two technical varieties have been successfully edited using CRISPR/Cas, while from the elite list of economically important grape varieties, only one variety, the white grape ‘Chardonnay’, has been edited in 8 years.

In 2016, it was shown that RNAi-mediated silencing of the susceptibility S gene *MLO7* significantly increased the resistance of grapevines to powdery mildew [10]. In the same year, another paper was published on the CRISPR/Cas editing of *MLO7*, in which the authors showed that knocking out this gene significantly reduced the susceptibility of grapevines to powdery mildew [50]. Therefore, in our work, we also focused on solving the most serious problem in vineyards worldwide: the susceptibility of “noble” grape varieties to fungal pathogens. To validate the editing techniques, we chose the target gene *MLO7*.

Despite the fact that work on microclonal propagation of grapes began 60 years ago, we were also required to significantly modify existing decontamination protocols and in vitro micropropagation procedures for technical grape varieties. This was necessary because the commonly used hypochlorite decontamination protocols were not as effective with plant material obtained from the field collections of grapes used. As a result, we have developed a new method for sterilising explants using a polyhexamethylene guanidine hydrochloride (PHMG), which had not previously been used in plant biotechnology, and also created a new one-step method for micropropagating grapes in vitro on PRM media.

In order to visually demonstrate the differences in metabolism and requirements of white and black varieties from the “Nobel” list, we carried out a visual experiment that illustrated the reliable differences in negative phenotypic manifestations when cultivated on different media used and recommended for working with grapes. The results obtained illustrate the differences in metabolism and requirements of different grape varieties. They show that the 1M–4M media used for microclonal propagation of grapes [71,72] were not the best choice for microclonal propagation of ‘Merlot’, ‘Malbec’, ‘Chardonnay’, and ‘Riesling’ varieties. The use of a new approach to microclonal propagation and cultivation of explants on PRM medium with charcoal is the only universal medium shown in our experiments to be suitable for a wide range of technical and table grape varieties.

In order to determine the metabolic characteristics of grape cells during the activation of the plant immune response to a pathogen, we also carried out a characterisation of the changes in phenolic and peroxide compounds of the technical black grape variety ‘Merlot’ when cultivated with chitosan as a pathogen infection model. A new approach was developed using chitosan as an inducer of the plant’s natural response to biotic stress. This helped to reduce the production of phenolic metabolites, making experiments with metabolic grape cell cultures more predictable and manageable.

In this study, during the CRISPR/Cas genome editing of the black grape variety ‘Merlot’, we observed significant differences in the frequencies of nonsense, missense, and synonymous mutations in the edited callus cells, which varied by two to three orders of magnitude compared with control samples transfected with vectors without guide RNAs. However, indel mutations, as the main class of mutations characteristic of editing, were not identified when using either the pKSE401 editing vector [69], the grape promoters editing vector for shRNA and Cas9 expression, or with the editing vector with a fluorescent peroxide sensor as a reporter. Only by modifying the cultivation and selection strategies, including the addition of chitosan as an inducer of the plant pathogen immune response, as well as additional washes and passages on media containing PVP, charcoal, and chitosan, were we able to increase the likelihood of successful CRISPR/Cas9 editing or the likelihood of selecting edited cells. According to other research, these edited cells should be characterised by an increase in the synthesis of peroxide compounds when in contact with the pathogen [58]. Thus, the use of the newly described strategy could select edited and potentially fungus-resistant grape forms by sorting explants by HyPer fluorescence level. This new approach may be particularly valuable in multiplex editing of grape and other plant crops.

One of the possible reasons for the low efficiency of editing using expression vector systems on the ‘Merlot’ variety may be the higher content of phenolic compounds, especially resveratrol, in red and black grape varieties. In 2017, Li et al. published a paper demonstrating an increase in the homologous repair (HDR) frequency and a decrease in repair frequencies by NHEJ when resveratrol was added to cell cultures. Resveratrol reduces the expression of *LIG4*, *PRKDC*, *KU70*, and *KU80* in the NHEJ pathway [73]. Thus, resveratrol may promote HDR development through its inhibitory effect on the NHEJ process. Over the last five years, several studies have also been published confirming the effect of resveratrol on the expression and activity of various components of the DNA repair and recombination systems in mammalian cells, suggesting the possibility of the existence of similar mechanisms with homologous genes and proteins in plants [74,75].

Undoubtedly, the mechanisms by which phenolic metabolites influence cell repair systems are extremely important to study, not only from a practical point of view, since we can potentially induce the desired repair pathway in cells during editing simply by adding metabolites such as resveratrol to the culture medium, but also because it makes us look at the explanation of the so-called ‘French paradox’ and treat unique cultures of metabolic plants that have been grown alongside humans for so long with a little more awareness.

An interesting indirect confirmation of the hypothesis that the efficiency of CRISPR/Cas editing is limited by phenolic metabolites is the fact that the first edited variety of the ‘Nobel’ dozen was ‘Chardonnay’.

It has been shown that the ‘Chardonnay’ is derived from another elite variety, ‘Pinot Noir’; the divergence occurred due to a mutation in the *VvMYBA1*/*A2* gene, resulting in the silencing of genes involved in the biosynthesis of flavonoids and anthocyanins [76]. In hops, for example, it has been shown that the constitutive expression of the TF HlMYB7 leads to the repression of flavonoid biosynthesis genes, whereas increased expression of the repressor HlMYB7 R2R3-MYB leads to increased accumulation of flavonoids in plant cells [77]. Similar results targeting TFs of the MYB family have been obtained in apple, rice, tomato, grape, persimmon, tea, strawberry, tobacco, and other plants, with changes in anthocyanin and flavonoids content in the modified plants [78,79,80,81,82,83,84,85,86,87,88,89]. It is known that the content of phenolic compounds in white grape varieties is one to two orders of magnitude lower than in black varieties (Database on Polyphenol Content in Foods—Phenol-Explorer).

‘Merlot’ is an economically important grape variety from the Bordeaux region of France. Today, ‘Merlot’ is grown in 37 countries around the world and is the second most widely planted grape variety in the world, after ‘Cabernet Sauvignon’ (https://www.oiv.int/public/medias/5888/endistribution-of-the-worlds-grapevine-varieties.pdf accessed on 24 July 2024). Developing pathogen-resistant varieties from the ‘Nobel’ list is an extremely important task for modern plant biotechnology. This work is the first to successfully edit the ‘Merlot’ variety and to develop a strategy to increase the probability of selecting fungus-resistant economically important grape varieties.

## 4. Materials and Methods

### 4.1. Materials

#### 4.1.1. Bacterial Strains

NEB-stable (NEB, Ipswich, MA, USA) *Escherichia coli* strain was used for plasmid amplification. For *Agrobacterium* transformation and plant transfection, strain EHA105 (from Professor, Dr. Ludmila A. Lutova of St. Petersburg State University) was used.

#### 4.1.2. Plant Materials

In this study, technical grape varieties ‘Malbec’, ‘Merlot’, ‘Chardonnay’ and ‘Riesling’ from the collection of the “All-Russian National Research Institute of Viticulture and Oenology “Magarach” were used.

#### 4.1.3. Editing Vectors

The pKSE401 plasmid was obtained from Dr. Yulia Ukhatova of the ‘N.I. Vavilov All-Russian Research Institute of Plant Genetic Resources (VIR)’. For the construction of new editing vectors, the following vectors were used: pGEM–T Easy (Promega, Madison, WI, USA), Gateway^®^ HyPer–AS entry clone (Evrogen, Moscow, Russia), pH_35S/mEGFP (from Mathias Pribil (Addgene plasmid # 135321; http://n2t.net/addgene:135321 accessed on 12 December 2021; RRID:Addgene_135321); pTF-FLPe (from James Birchler (Addgene plasmid # 139672; http://n2t.net/addgene:139672 accessed on 12 December 2021; RRID:Addgene_139672)) were used. The Gmhsp17.5-E promoter and p2a linker sequences were synthesised by Skygen (Moscow, Russia).

#### 4.1.4. Primers

The primers used in this work were selected using the online tools “In-Fusion Cloning Primer Design Tool” (https://www.takarabio.com/learning-centers/cloning/primer-design-and-other-tools accessed on 17 December 2021), OligoAnalyzer ™ Tool (https://eu.idtdna.com/pages/tools/oligoanalyzer accessed on 17 December 2021), BLAST-primer (https://www.ncbi.nlm.nih.gov/tools/primer-blast/ accessed on 20 December 2021) and SnapGene 6.0 software (www.snapgene.com accessed on 21 January 2022). The sequences of the most important primers are listed in Table S1.

#### 4.1.5. Enzymes and Kits

Enzymes (restriction endonucleases; T4-ligase; rSAP; T4-PNK; Q5 polymerase; DNA polymerase I, Large (Klenow) Fragment) (NEB, Ipswich, MA, USA) were used for all cloning steps. Plasmid DNA was purified using the kits “Plasmid Miniprep”, “Plasmid Midiprep” (Evrogen, Moscow, Russia), and “Plasmid mini/midi Kits” (QIAGEN Sciences Inc., Germantown, MD, USA). DNA fragments from agarose gels were purified using “Cleanup Mini” kits (Evrogen, Moscow, Russia). ScreenMix (Evrogen, Moscow, Russia) was used for PCR screening.

Genomic DNA from grape leaves and calli was isolated using the Plant RNA/DNA Purification Kit (Norgen Biotek Corp., Thorold, ON, Canada) or using CTAB buffer (2% CTAB; 1% PVP; 20 mM EDTA; 100 mM Tris HCl; 1.4 M NaCl) (OPS Diagnostics LLC, Lebanon, NJ, USA).

#### 4.1.6. Agarose Gels

For DNA agarose gel electrophoresis, 0.7–1% agarose TopVision (Thermo Scientific, Waltham, MA, USA) gels and TAE buffer (Thermo Scientific, Waltham, MA, USA) were used.

### 4.2. Methods

#### 4.2.1. Decontamination and Cultivation of Plants

Apical and axillary buds with one leaf bud were used to initiate in vitro culture. These samples were collected from the field collection of the All-Russian National Scientific Research Institute of Viticulture and Oenology “Magarach”. Two approaches were used for sterilisation: (1) surface sterilisation for 15 min with sodium hypochlorite (1.5% available chlorine) in bleach (Procter & Gamble, Moscow, Russia) containing a few drops of Tween 20 as a wetting agent in Falcon tubes by gentle rotation at 100 rpm, followed by four washes with sterile distilled water, drying through sterile filter paper; (2) surface sterilisation by washing with a 10% solution of polyhexamethylene guanidine hydrochloride (PHMG) (Soft Protector, Moscow, Russia) was carried out for 20 min in Falcon tubes by gentle rotation at 100 rpm, followed by four washes with sterile distilled water, drying with sterile filter paper, and then planting on the initial culture medium.

The cultivation of the plants was carried out in climatic chambers with a light intensity of 70 µmol m⁻^2^ s⁻^1^ 25 °C with a light regime of 16 h of light and 8 h of darkness.

#### 4.2.2. Micropropagation of Plants

Media recommended for the microclonal propagation of grapevine varieties were used [71,72,90]. In our previous work, the MS medium without hormones was also used for the initiation phase, but unfortunately, all the tested media were not suitable for the microclonal propagation of the elite technical grape varieties ‘Malbec’, ‘Merlot’ and ‘Riesling’, so in this research, we used the following culture media [91]: microelements (Sigma-Aldrich, Steinheim am Main, Germany): CuSO_4_ × 5H_2_O 0.25 mg/L; H_3_BO_3_ 6.2 mg/L; MnSO_4_ × H_2_O 22.3 mg/L; Na_2_MoO_4_ × 2H_2_O 0.25 mg/L; and ZnSO_4_ × 7H_2_O 8.6 mg/L; macroelements (Sigma-Aldrich, Steinheim am Main, Germany): CaCl_2_ × 2H_2_O 96 mg/L; Ca(NO_3_)_2_ × 4H_2_O 471.26 mg/L; KH_2_PO_4_ 170 mg/L; K_2_SO_4_ 990 mg/L; MgSO_4_ 180.54 mg/L; FeSO_4_ × 7H_2_O 27.8 mg/L; Na_2_EDTA × 2H_2_O 37.26 mg/L; and NH_4_NO_3_ 400 mg/L; vitamins (Sigma-Aldrich, Steinheim am Main, Germany): nicotinic acid 0.5 mg/L; pyridoxine HCl 0.5 mg/L; thiamine HCl 1 mg/L; 100 mg/L vitamin C (Armavir Biofactory FKP, Armavir, Russia) [38,39]: 0.2 mg/L 2-naphthoxyacetic acid (NAA) (Sigma-Aldrich, Steinheim am Main, Germany); and 0.2 mg/L 6-benzyladenine (6-BAP) (Sigma-Aldrich, Steinheim am Main, Germany); glycine 2 mg/L; myo-inositol 100 mg/L; activated charcoal 2 g/L, pH 5.75, agar 4% (Biolot, Saint-Petersburg, Russia), and phytagel 2% (Sigma-Aldrich, Steinheim am Main, Germany).

#### 4.2.3. Callus Induction

For callus induction, healthy leaves and cuttings from in vitro plants and MS media consisting of half-strength and 50% of the inorganic nitrate salts of MS 1 mg/L 6-BAP (Sigma-Aldrich, Steinheim am Main, Germany), 0.1 mg/L 4-(3-Indolyl) butyric acid (IBA) (Sigma-Aldrich, Steinheim am Main, Germany) or half-strength and 50% of the inorganic nitrate salts of MS with 2,4-dichlorophenoxyacetic acid (2.4 D) 9 mg/L (Phytotech Labs, Westfield, NJ, USA), 4.5 mg/L 6-BAP (Sigma-Aldrich, Steinheim am Main, Germany) were used.

#### 4.2.4. *Agrobacterium* Transformation and Transfection

The preparation of competent cells and agrobacterial transformation were carried out according to the freeze–thaw method [92].

Liquid or solid YEP medium was used for the selection and cultivation of the EHA105 strain. YEP consists of 10 g/L bactotryptone (Difco, Life Technologies Corp., Detroit, MI, USA), 10 g/L yeast extract (Difco, Life Technologies Corp., Detroit, MI, USA), 5 g/L NaCl 10 g/L agar (Biolot, Saint-Petersburg, Russia) (if required for solid medium), and an addition of 50 µg/L kanamycin monosulfate (Sigma-Aldrich, Steinheim am Main, Germany) (if required for selection).

For cocultivation, callus decontamination, and selection, 1/2MD medium was used: half-strength and 50% of the inorganic nitrate salts of MS salts and vitamins, 1 mL/L 2, 4-D stock solution (final concentration is 2 µM), 30 g/L sucrose, 4% agar (Biolot, Russia) (for solid medium), 0.5 g/L glutamine (Phytotech Labs, Westfield, NJ, USA), and 1 g/L casamino acid (Phytotech Labs, Westfield, NJ, USA). The cocultivation medium was supplemented with acetosyringone (final concentration 100 µM) (Phytotech Labs, Westfield, NJ, USA) and decontamination medium was supplemented with 500 mg/L Cefotaxime (Phytotech Labs, Westfield, NJ, USA) [93]. Callus pieces were transferred to fresh media every week, rinsing the explants with decontamination medium supplemented with 1 g/L chitosan (Vitauct, Abadzekhskaya, Russia).

Optimised media for callus preculture of callus coculture with *Agrobacterium* suspension stages: 1/2MD supplemented with polyvinylpyrrolidone K30 (PVP) (TNJ, Shanghai, China) 0.5 g/L. Optimised preculture media was also supplemented with chitosan (Vitauct, Abadzekhskaya, Russia) 1 g/L.

#### 4.2.5. Plasmid Construction

Cloning of guide RNA expression sequences into the editing vectors was carried out according to the protocol described in the article by Xing using golden gate cloning of a fragment of renatured primers with artificially generated protruding ends, complementary to the ends formed after hydrolysis of the editing vector by BsaI (NEB, Ipswich, MA, USA) restriction endonuclease.

To select the most promising guide RNA for editing the target gene (*MLO7*), the following online tools were used: CRISPR-P 2.0 (https://crispr.hzau.edu.cn/cgi-bin/CRISPR2/CRISP, accessed on 26 January 2022), CRISPOR (https://crispor.tefor.net/ accessed on 26 January 2022), SYNTHEGO (https://design.synthego.com/#/ accessed on 30 January 2022), and CAS-OFFinder (http://www.rgenome.net/cas-offinder/ accessed on 30 March 2022).

To increase regeneration and the possibility of selecting transformants without the use of antibiotics, but by the fluorescence of the reporter protein GFP, the selective marker NeoR/KanR (pKSE401) was replaced by a chimeric selective marker GFP-p2a-HygR. The chimeric construct was assembled by fusion PCR. The GFP and HygR sequences were amplified on the pH_35S/mEGFP vector template, and the p2A sequence was amplified on the template sequence synthesised by Skygen LLC (Moscow, Russia). Also, FRT (FLP flippase recognition sequences) and the LB repeat (required for the insertion of the sequence into the plant genome during *Agrobacterium*-mediated transfection) were also introduced downstream of the chimeric marker. PolyA ends were added to the chimeric PCR product using Taq polymerase (Evrogen, Moscow, Russia). The PCR product was purified from the reaction mixture, cloned into pGEM-T Easy plasmid, and plasmid DNA was isolated from AmpR NeB-stable *E. coli* clones and verified by restriction endonucleases NotI and BamHI hydrolysis and Sanger sequencing. After confirming the correct assembly of the GFP-p2A-HygR chimeric sequence, the pGEM-T-Easy-eGFP-p2A-HygR intermediate construct was hydrolysed by restriction endonucleases NcoI and SacII, and the 2475 bp fragment was purified from a 0.7% agarose gel and ligated into the pKSE401/NcoI, SacII, and rSAP vector (15,063 bp). This produced the pHSE-EGFP vector.

In the next step, a sequence encoding the flippase FLP was inserted into the modified core vector pHSE-EGFP under the inducible heat of the Gmhsp17.5-E promoter to allow the excision of the agrobacterial vector inserts by FRT recognition sites, which led to the selection of edited non-transgenic plants in the future final stage of the research. FLP expression will be induced by heat after editing the targets with the Cas9 nuclease: the flippase will cut out the insertion of the *Agrobacterium* vector insertion at the FRT sequences, allowing the selection of the edited non-transgenic plant [70]. The HSP-FLP PCR product (2.9 kb) was hydrolysed at the flanking recognition sites SacI and StuI, and ligated with the pHSE-EGFP-HygR/SbfI, EcoRI vector (11.7 kb). This resulted in the vector pHSE-EGFP-FLP vector.

To optimise the core vector pHSE-EGFP-FLP, promoter replacement for Cas9 and sgRNA expression was performed [59]. For this purpose, the UBQ2 P and U6-2P promoter sequences were amplified using the ‘Riesling Elute’ grape genomic DNA as a template. A fragment containing the VvU6-26 and VvUBQ promoters was constructed by PCR using overlapping primers (Supplementary Table S1). The intermediate vector pHSE-EGFP-FLP was hydrolysed by SbfI to excise the CaMV 35S promoter region. The linearised and dephosphorylated vector without the CaMV 35S promoter sequence (19.1 kb) was prepared by isolation from the gel and ligation with the 9F-12R/SbfI, PstI fragment (1.2 kb) obtained by the hydrolysis of the intermediate construct pGEM-T Easy-9F-12R. After screening and sequence verification, the vector pHSE-EGFP-FLP-VvUBQ2 was obtained.

The VvU6-26P promoter was introduced by hydrolysis of the intermediate vector pHSE-EGFP-FLP-VvUBQ2 with endonucleases PmeI, BsaI, and the purified fragments of 18.4 kb and 1.3 kb were ligated with a PCR product (801 bp) amplified on the template of the pGEM-T-Easy-1F_FRT-8R intermediate vector hydrolysed by BsaI restriction endonuclease. After NEB-stable transformation, Km+ Str+ colonies were screened by PCR. The correctness of the vector assembly was confirmed by Sanger sequencing. The resulting vector was pHSE-EGFP-FLP-VvUBQ2-VvU6.

To integrate the HyPer reporter into the editing vector, PCR mutagenesis of two NcoI sites was a necessary step. Hyper is a genetically encoded fluorescent sensor of intracellular hydrogen peroxide [93]. The commercial vector Gateway^®^ HyPer-AS entry clone (Evrogen, Moscow, Russia) was used as a template. Hyper PCR product was ligated into intermediate vector pGEM-T-Easy; then, it was hydrolysed with NcoI, SacII endonucleases, and a fragment (2056 bp) with the HyPer sequence, flanked by NcoI and SacII sites, was isolated. The 2056 bp fragment, containing the sequence of mutagenised HyPer without recognition sites NcoI, CaMV polyA signal, FRT recognition signal, and LB repeat for integration into the plant genome during *Agrobacterium* transfection, was ligated to the pHSE-EGFP-FLP-VvUBQ2-VvU6/NcoI, SacII (18.041 kb) vector. After NEB stable transformation, Km+ Str+ colonies were screened by PCR. The correctness of the vector assembly was confirmed by Sanger sequencing. The new editing vector pHy-per-FLP-VvUBQ2-VvU6 was obtained (Genebank submission number: # 2854555).

#### 4.2.6. Editing Efficiency and Genotyping

Km+ callus cells (transfected with pKSE401-based editing vector), Hyg+ callus cells (transfected with pHSE-EGFP-based editing vector), or Hyper+ callus cells (transfected with pHyper-FLP-VvUBQ2-VvU6 based editing vector) were used for genomic DNA purification using the Norgen kit (Norgen, Thorold, ON, Canada). The prepared genomic DNA of transfectants (50–100 ng) was used as a template for the amplification of gene fragments containing target sites using high-fidelity Q5 polymerase (NEB, Ipswich, MA, USA) or Kapa3G plant polymerase (Roche Diagnostics, Indianapolis, IN, USA). Amplicons were sequenced using Sanger or MiSeq NGS, and sequences were analysed using the Synthego ICE [94] and Cas-Analyzer online tools [95]. Amplicon libraries from explants, transfected with vectors without guide RNAs were used as control samples to assess the level of spontaneous mutations in unedited genomes.

#### 4.2.7. Sequencing

Sanger sequencing was performed on an ABI 3500 Genetic Analyzer cycler (Applied Biosystems, Thermo Fisher Scientific, Waltham, MA, USA) using standard conditions and reagents recommended by the manufacturer Applied Biosystems (Applied Biosystems, Thermo Fisher Scientific, Waltham, MA, USA); PCR mixtures were purified using the BigDye XTerminator™ Purification Kit (Applied Biosystems, Thermo Fisher Scientific, Waltham, MA, USA).

Next-generation sequencing was performed using the Illumina method on the Mi-Seq platform. Gene amplicon fragmentation was performed on a Covais 220 Focused-ultrasonicator ultrasonic homogeniser using Covaris microTUBE-50 AFA fibre screw-cap tubes at a 75 W peak incident power, 20% duty factor; 1000 cycles per burst over 45 s. Sequencing libraries were prepared using Illumina TruSeq DNA PCR-Free kits, Illumina IDT for Illumina—TruSeq DNA UD Indexes (96 Indexes, 96 Samples), using a QuantStudio3 cycler (Applied Biosystems, Thermo Fisher Scientific, Waltham, MA, USA). Libraries were quantified on an Agilent TapeStation 4150 automated electrophoresis system (Agilent, Santa Clara, CA, USA) using D5000 High Sensitivity DNA kits (Agilent, Santa Clara, CA, USA) and the KAPA Library Quantification Kit (Roche, Indianapolis, IN, USA).

#### 4.2.8. Isolation of Protoplasts

Protoplasts were isolated following previously described optimised protocols [50,53], but with 0.2% macerozyme R-10, 0.75% cellulase R-10 in cell wall digestion enzyme solution mix, without vacuum infiltration, cultivation with enzymes in the dark, 19 °C, for 16 h, without rotation.

Protoplasts were isolated from 20-day-old young and healthy leaves micropropagated in vitro. Approximately 40 young leaves per 50 mL of Buffer C (20 mM MES, 0.5 M mannitol, 20 mM KCl, and 10 mM CaCl_2_, 0.2% macerozyme R-10 (Duchefa Biochemie, Haarlem, The Netherlands), 0.75% cellulase R-10 (Duchefa Biochemie, Haarlem, The Netherlands) were cut into 4–5 mm pieces and immersed in a mixture of enzyme solutions. No preliminary steps of plasmolysis or vacuum infiltration of the leaves with enzymes were performed.

After the enzymatic digestion step, the protoplasts were filtered through a 70 μm cell strainer (Corning, New York, NY, USA) with rinsing of the residue (debris and protoplasts) with 50 mL of wash solution W5 (5 mM glucose, 2 mM MES, pH 5.7, 154 mM NaCl, 125 mM CaCl_2_, and 5 mM KCl). The resulting suspension was centrifuged at 100× *g* for 5 min, and the supernatant was carefully collected without disturbing the protoplast pellet. The collected cells were suspended in W5 solution. Using a wide-bore pipette tip, the protoplast suspension was slowly layered onto 21% sucrose solution, followed by centrifugation. The interphase containing viable and healthy protoplasts was collected using a wide-bore pipette tip. The protoplasts were then suspended in 50 mL of the W5 solution and incubated for 1 h at 4 °C, 1 h/overnight at 4 °C. The protoplasts were centrifuged, the supernatant was discarded, and the protoplasts were resuspended in a MMG solution (0.5 M mannitol, 4 mM MES, pH 5.7, and 15 mM MgCl_2_). The concentration of the resulting protoplast suspension was determined using a Nikon Eclipse Ti-2 microscope (Nicon Corp., Tokyo, Japan) and Goryaev’s chamber (MiniMed, Suponevo, Russia) or a Countess 3 (Invitrogen, Carlsbad, CA, USA) automated cell counter. Viable cells were perfectly round with no visible defects on the plasma membrane and were not stained with 0.04% trypan blue [96].

#### 4.2.9. Measurement of Phenolic Content

One week after incubation in media with chitosan, aliquots of callus suspensions were selected, the cells were pelleted by centrifugation at 6000 rpm for 3 min, the supernatant medium was collected, the wet sediments were weighed, an equal volume of absolute ethyl alcohol (AquaM, Moscow, Russia) was added, and they were then frozen in liquid nitrogen. After thawing the samples, the suspensions were vortexed and extracted in a water bath at 45 °C for 45 min. After incubation, the extracts containing cell debris were centrifuged at 16,000 rpm for 2 min, the alcohol extracts were transferred to new tubes, and the reaction mixtures were mixed to determine the concentration of the total phenolic metabolite fraction: 25 µL of alcohol extract, 25 µL of Folin’s reagent (Biorad, Hercules, CA, USA), 50 µL of Na_2_CO_3_ (Sigma-Aldrich, Steinheim am Main, Germany), and 50 µL of mQ water; the samples were then incubated for 1 h at room temperature and the absorbance was measured at 700 nm on a NanoDrop OneC spectrophotometer (Thermo Fisher Scientific, Waltham, MA, USA). The methodology for the measurement of total phenolic metabolite content in plant extracts was based on reduced phosphotungstic and phosphomolybdic heteropolyacids of different modifications of Folin’s reagents in alkaline medium. Phenolic compounds refer to a blue complex (tungsten blue or heteropoly blue), the colour of which is proportional to the amount of phenolic compounds present. The total phenolic metabolite values were calculated as the equivalent to one microgram of resveratrol (Solgar, Leonia, NJ, USA) [97].

#### 4.2.10. Assessment of Intracellular Peroxide Compound Levels

Callus suspensions were transferred to a 48-well culture plate in media with and without chitosan, and fluorescence measurements and detection of changes in intracellular peroxide compound levels were carried out for three days using the Incucyte Live Cell Imaging and Analysis System (Sartorius, Göttingen, Germany). The level of fluorescence correlates with the concentration of peroxide compounds in the callus cells due to the HyPer peroxide sensor, which is present as a reporter in the editing vector pHyPer-FLP-VvUBQ2-VvU6 [68].

#### 4.2.11. Statistical Analysis

Statistical analyses of the data were performed using online tools: (https://statisty.app// accessed on 12 December 2023; https://epitools.ausvet.com.au/ztesttwo/ accessed on 12 December 2023) and Excel tools with statistical add ins. A two-tailed-z-test, Mann–Whitney test, and Fisher’s test were used [98,99,100].

## Data Availability

Data are contained within the article or Supplementary Materials.

## Supplementary Materials

The following supporting information can be downloaded at: https://www.mdpi.com/article/10.3390/ijms251810011/s1.

## References

[1] McGovern P. (2003). Ancient Wine: The Search for the Origins of Viniculture.

[2] Barbalho S., Ottoboni A., Fiorini A., Landgraf Guiguer E., Nicolau C., Goulart R., Flato U. (2020). Grape Juice or Wine: Which Is the Best Option?. Crit. Rev. Food Sci. Nutr..

[3] Cerezo A., Labrador M., Gutiérrez A., Hornedo Ortega R., Troncoso A., Garcia-Parrilla M. (2019). Anti-VEGF Signalling Mechanism in HUVECs by Melatonin, Serotonin, Hydroxytyrosol and Other Bioactive Compounds. Nutrients.

[4] Copetti C., Franco F.W., Machado E.D.R., Soquetta M.B., Quatrin A., Ramos V.M. (2018). Acute Consumption of Bordo Grape Juice and Wine Improves Serum Antioxidant Status in Healthy Individuals and Inhibits Reactive Oxygen Species Production in Human Neuron-Like Cells. J. Nutr. Metab..

[5] Toaldo I.M., Cruz F.A., da Silva E.L., Bordignon-Luiz M.T. (2016). Acute Consumption of Organic and Conventional Tropical Grape Juices (*Vitis labrusca* L.) Increases Antioxidants in Plasma and Erythrocytes, but Not Glucose and Uric Acid Levels, in Healthy Individuals. Nutr. Res..

[6] Hasan M., Bae H. (2017). Overview of Stress-Induced Resveratrol Synthesis in Grapes: Perspectives for Resveratrol-Enriched Grape Products. Molecules.

[7] Schwager J., Richard N., Widmer F., Raederstorff D. (2017). Resveratrol Distinctively Modulates the Inflammatory Profiles of Immune and Endothelial Cells. BMC Complement. Altern. Med..

[8] Munir I., Yen H.-W., Baruth T., Tarkowski R., Azziz R., Magoffin D.A., Jakimiuk A.J. (2005). Resistin Stimulation of 17α-Hydroxylase Activity in Ovarian Theca Cells in Vitro: Relevance to Polycystic Ovary Syndrome. J. Clin. Endocrinol. Metab..

[9] Cohen P., Bacilieri R., Ramos-Madrigal J., Privman E., Boaretto E., Weber A., Fuks D., Weiss E., Erickson-Gini T., Bucking S. (2023). Ancient DNA from a Lost Negev Highlands Desert Grape Reveals a Late Antiquity Wine Lineage. Proc. Natl. Acad. Sci. USA.

[10] Pessina S., Lenzi L., Perazzolli M., Campa M., Dalla Costa L., Urso S., Valè G., Salamini F., Velasco R., Malnoy M. (2016). Knockdown of MLO Genes Reduces Susceptibility to Powdery Mildew in Grapevine. Hortic. Res..

[11] Dries L., Hendgen M., Schnell S., Löhnertz O., Vortkamp A. (2021). Rhizosphere Engineering: Leading towards a Sustainable Viticulture?. OENO One.

[12] Velasco R., Zharkikh A., Troggio M., Cartwright D.A., Cestaro A., Pruss D., Pindo M., FitzGerald L.M., Vezzulli S., Reid J. (2007). A High Quality Draft Consensus Sequence of the Genome of a Heterozygous Grapevine Variety. PLoS ONE.

[13] Ramos M.J.N., Coito J.L., Faísca-Silva D., Cunha J., Costa M.M.R., Amâncio S., Rocheta M. (2020). Portuguese Wild Grapevine Genome Re-Sequencing (*Vitis vinifera sylvestris*). Sci. Rep..

[14] Bavaresco L. (2019). Impact of Grapevine Breeding for Disease Resistance on the Global Wine Industry. Acta Hortic..

[15] Ngou B.P.M., Ding P., Jones J.D.G. (2022). Thirty Years of Resistance: Zig-Zag through the Plant Immune System. Plant Cell.

[16] Boller T., Felix G. (2009). A Renaissance of Elicitors: Perception of Microbe-Associated Molecular Patterns and Danger Signals by Pattern-Recognition Receptors. Annu. Rev. Plant Biol..

[17] Guo J., Cheng Y. (2022). Advances in Fungal Elicitor-Triggered Plant Immunity. Int. J. Mol. Sci..

[18] Mittler R. (2002). Oxidative Stress, Antioxidants and Stress Tolerance. Trends Plant Sci..

[19] Zurbriggen M., Carrillo N., Hajirezaei M. (2010). ROS Signaling in the Hypersensitive Response: When, Where and What For?. Plant Signal Behav..

[20] Rkhaila A., Chtouki T., Erguig H., El Haloui N., Ounine K. (2021). Chemical Proprieties of Biopolymers (Chitin/Chitosan) and Their Synergic Effects with Endophytic Bacillus Species: Unlimited Applications in Agriculture. Molecules.

[21] Melotto M., Underwood W., Sheng Y.H. (2008). Role of Stomata in Plant Innate Immunity and Foliar Bacterial Diseases. Annu. Rev. Phytopathol..

[22] Sawinski K., Mersmann S., Robatzek S., Böhmer M. (2013). Guarding the Green: Pathways to Stomatal Immunity. Mol. Plant Microbe Interact..

[23] Chen L.-Q., Hou B.-H., Lalonde S., Takanaga H., Hartung M.L., Qu X.-Q., Guo W.-J., Kim J.-G., Underwood W., Chaudhuri B. (2010). Sugar Transporters for Intercellular Exchange and Nutrition of Pathogens. Nature.

[24] Wang K., Senthil-Kumar M., Ryu C.M., Kang L., Mysore K.S. (2012). Phytosterols Play a Key Role in Plant Innate Immunity against Bacterial Pathogens by Regulating Nutrient Efflux into the Apoplast. Plant Physiol..

[25] Mur L.A.J., Kenton P., Lloyd A.J., Ougham H., Prats E. (2008). The Hypersensitive Response; The Centenary Is upon Us but How Much Do We Know?. J. Exp. Bot..

[26] O’Brien J.A., Benková E. (2013). Cytokinin Cross-Talking during Biotic and Abiotic Stress Responses. Front. Plant Sci..

[27] Murphy C.M. (1999). Plant Products as Antimicrobial Agents. Clin. Microbiol. Rev..

[28] van Loon L.C., Rep M., Pieterse C.M.J. (2006). Significance of Inducible Defense-Related Proteins in Infected Plants. Annu. Rev. Phytopathol..

[29] Qiu W., Feechan A., Dry I. (2015). Current Understanding of Grapevine Defense Mechanisms against the Biotrophic Fungus (*Erysiphe necator*), the Causal Agent of Powdery Mildew Disease. Hortic. Res..

[30] Pavan S., Schiavulli A., Appiano M., Marcotrigiano A., Cillo F., Visser R., Bai Y., Lotti C., Ricciardi L. (2011). Pea Powdery Mildew Er1 Resistance Is Associated to Loss-of-Function Mutations at a MLO Homologous Locus. Theor. Appl. Genet..

[31] Pavan S., Jacobsen E., Visser R.G.F., Bai Y. (2010). Loss of Susceptibility as a Novel Breeding Strategy for Durable and Broad-Spectrum Resistance. Mol. Breed..

[32] Steinbrenner A.D., Goritschnig S., Krasileva K.V., Schreiber K.J., Staskawicz B.J. (2012). Effector Recognition and Activation of the Arabidopsis Thaliana NLR Innate Immune Receptors. Cold Spring Harb. Symp. Quant. Biol..

[33] Jørgensen J.H. (2004). Discovery, Characterization and Exploitation of Mlo Powdery Mildew Resistance in Barley. Euphytica.

[34] Consonni C., Humphry M.E., Hartmann H.A., Livaja M., Durner J., Westphal L., Vogel J., Lipka V., Kemmerling B., Schulze-Lefert P. (2006). Conserved Requirement for a Plant Host Cell Protein in Powdery Mildew Pathogenesis. Nat. Genet..

[35] Bai Y., Pavan S., Zheng Z., Zappel N.F., Reinstädler A., Lotti C., De Giovanni C., Ricciardi L., Lindhout P., Visser R. (2007). Naturally Occurring Broad-Spectrum Powdery Mildew Resistance in a Central American Tomato Accession Is Caused by Loss of Mlo Function. Mol. Plant Microbe Interact..

[36] Zheng Z., Nonomura T., Appiano M., Pavan S., Matsuda Y., Toyoda H., Wolters A.M.A., Visser R.G.F., Bai Y. (2013). Loss of Function in *Mlo* Orthologs Reduces Susceptibility of Pepper and Tomato to Powdery Mildew Disease Caused by *Leveillula taurica*. PLoS ONE.

[37] Gadoury D.M., Cadle-Davidson L.A., Wilcox W.F., Dry I.B., Seem R.C., Milgroom M.G. (2012). Grapevine Powdery Mildew (*Erysiphe necator*): A Fascinating System for the Study of the Biology, Ecology and Epidemiology of an Obligate Biotroph. Mol. Plant Pathol..

[38] Dufour M.-C., Fontaine S., Montarry J., Corio-Costet M.-F. (2011). Assessment of Fungicide Resistance and Pathogen Diversity in Erysiphe Necator Using Quantitative Real-Time PCR Assays. Pest. Manag. Sci..

[39] Feechan A., Jermakow A., Torregrosa L., Panstruga R., Dry I. (2009). Identification of Grapevine MLO Gene Candidates Involved in Susceptibility to Powdery Mildew. Funct. Plant Biol..

[40] Winterhagen P., Howard S., Qiu W., Kovacs L. (2008). Transcriptional Up-Regulation of Grapevine MLO Genes in Response to Powdery Mildew Infection. Am. J. Enol. Vitic..

[41] Chen Z., Kloek A.P., Boch J., Katagiri F., Kunkel B.N. (2000). The Pseudomonas Syringae AvrRpt2 Gene Product Promotes Pathogen Virulence from Inside Plant Cells. Mol. Plant Microbe Interact..

[42] Massonnet M., Cochetel N., Minio A., Vondras A.M., Lin J., Muyle A., Garcia J.F., Zhou Y., Delledonne M., Riaz S. (2020). The Genetic Basis of Sex Determination in Grapes. Nat. Commun..

[43] Töpfer R., Trapp O. (2022). A Cool Climate Perspective on Grapevine Breeding: Climate Change and Sustainability Are Driving Forces for Changing Varieties in a Traditional Market. Theor. Appl. Genet..

[44] Grassi F., De Lorenzis G. (2021). Back to the Origins: Background and Perspectives of Grapevine Domestication. Int. J. Mol. Sci..

[45] Zhu H., Li C., Gao C. (2020). Applications of CRISPR–Cas in Agriculture and Plant Biotechnology. Nat. Rev. Mol. Cell Biol..

[46] Fizikova A., Tikhonova N., Ukhatova Y., Ivanov R., Khlestkina E. (2021). Applications of CRISPR/Cas9 System in Vegetatively Propagated Fruit and Berry Crops. Agronomy.

[47] Jogam P., Sandhya D., Alok A., Peddaboina V., Allini V.R., Zhang B. (2022). A Review on CRISPR/Cas-Based Epigenetic Regulation in Plants. Int. J. Biol. Macromol..

[48] Qi Q., Hu B., Jiang W., Wang Y., Yan J., Ma F., Guan Q., Xu J. (2023). Advances in Plant Epigenome Editing Research and Its Application in Plants. Int. J. Mol. Sci..

[49] Ren C., Liu X., Zhang Z., Wang Y., Duan W., Li S., Liang Z. (2016). CRISPR/Cas9-Mediated Efficient Targeted Mutagenesis in Chardonnay (*Vitis vinifera* L.). Sci. Rep..

[50] Malnoy M., Viola R., Jung M.-H., Koo O., Kim S., Kim J.-S., Velasco R., Kanchiswamy C. (2016). DNA-Free Genetically Edited Grapevine and Apple Protoplast Using CRISPR/Cas9 Ribonucleoproteins. Front. Plant Sci..

[51] Nakajima I., Ban Y., Azuma A., Onoue N., Moriguchi T., Yamamoto T., Toki S., Endo M. (2017). CRISPR/Cas9-Mediated Targeted Mutagenesis in Grape. PLoS ONE.

[52] Wang X., Tu M., Wang D., Liu J., Li Y., Li Z., Wang Y., Wang X. (2018). CRISPR/Cas9-Mediated Efficient Targeted Mutagenesis in Grape in the First Generation. Plant Biotechnol. J..

[53] Osakabe Y., Liang Z., Ren C., Nishitani C., Osakabe K., Wada M., Komori S., Malnoy M., Velasco R., Poli M. (2018). CRISPR–Cas9-Mediated Genome Editing in Apple and Grapevine. Nat. Protoc..

[54] Sun X., Zhang S., Li X., Zhang X., Wang X., Wang L., Li Z., Wang X. (2020). A MADS-Box Transcription Factor from Grapevine, VvMADS45, Influences Seed Development. Plant Cell Tissue Organ. Cult..

[55] Sunitha S., Rock C.D. (2020). CRISPR/Cas9-Mediated Targeted Mutagenesis of TAS4 and MYBA7 Loci in Grapevine Rootstock 101-14. Transgenic Res..

[56] Li M.Y., Jiao Y.T., Wang Y.T., Zhang N., Wang B.B., Liu R.Q., Yin X., Xu Y., Liu G.T. (2020). CRISPR/Cas9-Mediated *VvPR4b* Editing Decreases Downy Mildew Resistance in Grapevine (*Vitis vinifera* L.). Hortic. Res..

[57] Ren C., Guo Y., Kong J., Lecourieux F., Dai Z., Li S., Liang Z. (2020). Knockout of *VvCCD8* Gene in Grapevine Affects Shoot Branching. BMC Plant Biol..

[58] Wan D.Y., Guo Y., Cheng Y., Hu Y., Xiao S., Wang Y., Wen Y.Q. (2020). CRISPR/Cas9-Mediated Mutagenesis of *VvMLO3* Results in Enhanced Resistance to Powdery Mildew in Grapevine (*Vitis vinifera*). Hortic. Res..

[59] Ren C., Liu Y., Guo Y., Duan W., Fan P., Li S., Liang Z. (2021). Optimizing the CRISPR/Cas9 System for Genome Editing in Grape by Using Grape Promoters. Hortic. Res..

[60] Olivares F., Loyola R., Olmedo B., Miccono M.D., Aguirre C., Vergara R., Riquelme D., Madrid G., Plantat P., Mora R. (2021). CRISPR/Cas9 Targeted Editing of Genes Associated with Fungal Susceptibility in *Vitis vinifera* L. cv. Thompson Seedless Using Geminivirus-Derived Replicons. Front. Plant Sci..

[61] Scintilla S., Salvagnin U., Giacomelli L., Zeilmaker T., Malnoy M.A., van der Voort J., Moser C. (2022). Regeneration of Non-Chimeric Plants from DNA-Free Edited Grapevine Protoplasts. Front. Plant Sci..

[62] Ren C., Gathunga E.K., Li X., Li H., Kong J., Dai Z., Liang Z. (2023). Efficient Genome Editing in Grapevine Using CRISPR/LbCas12a System. Mol. Hortic..

[63] Villette J., Lecourieux F., Bastiancig E., Héloir M.-C., Poinssot B. (2024). New Improvements in Grapevine Genome Editing: High Efficiency Biallelic Homozygous Knock-out from Regenerated Plantlets by Using an Optimized ZCas9i. Plant Methods.

[64] Gambino G., Nuzzo F., Moine A., Chitarra W., Pagliarani C., Petrelli A., Boccacci P., Delliri A., Velasco R., Nerva L. (2024). Genome Editing of a Recalcitrant Wine Grape Genotype by Lipofectamine-Mediated Delivery of CRISPR/Cas9 Ribonucleoproteins to Protoplasts. Plant J..

[65] Yang Y., Wheatley M., Meakem V., Galarneau E., Gutierrez B., Zhong G.-Y. (2024). Editing *VvDXS1* for the Creation of Muscat Flavour in *Vitis vinifera* cv. Scarlet Royal. Plant Biotechnol. J..

[66] Cui Z.-H., Bi W.-L., Hao X.-Y., Li P.-M., Duan Y., Walker M.A., Xu Y., Wang Q.-C. (2017). Drought Stress Enhances Up-Regulation of Anthocyanin Biosynthesis in Grapevine Leafroll-Associated Virus 3-Infected in Vitro Grapevine (*Vitis vinifera*) Leaves. Plant Dis..

[67] Meggio F., Zarco-Tejada P.J., Núñez L.C., Sepulcre-Cantó G., González M.R., Martín P. (2010). Grape Quality Assessment in Vineyards Affected by Iron Deficiency Chlorosis Using Narrow-Band Physiological Remote Sensing Indices. Remote Sens. Environ..

[68] Belousov V.V., Fradkov A.F., Lukyanov K.A., Staroverov D.B., Shakhbazov K.S., Terskikh A.V., Lukyanov S. (2006). Genetically Encoded Fluorescent Indicator for Intracellular Hydrogen Peroxide. Nat. Methods.

[69] Xing H.-L., Dong L., Wang Z.-P., Zhang H.-Y., Han C.-Y., Liu B., Wang X.-C., Chen Q.-J. (2014). A CRISPR/Cas9 Toolkit for Multiplex Genome Editing in Plants. BMC Plant Biol..

[70] Pompili V., Dalla Costa L., Piazza S., Pindo M., Malnoy M. (2020). Reduced Fire Blight Susceptibility in Apple Cultivars Using a High-Efficiency CRISPR/Cas9-FLP/FRT-Based Gene Editing System. Plant Biotechnol. J..

[71] Beza K., Feyissa T., Bedada G. (2017). In Vitro Micropropagation of Grape Vine (*Vitis vinifera* L.) from Nodal Culture. Afr. J. Biotechnol..

[72] Mostafa F., Shaaban M., Elazab D., Kamel M. (2015). In Vitro Propagation of Four Grape Cultivars. Assiut J. Agric. Sci..

[73] Li G., Xianwei Z., Zhong C., Mo J., Quan R., Yang J., Liu D., Li Z., Yang H., Wu Z. (2017). Small Molecules Enhance CRISPR/Cas9-Mediated Homology-Directed Genome Editing in Primary Cells. Sci. Rep..

[74] Ma F., Ma Y., Liu K., Gao J., Li S., Sun X., Li G. (2023). Resveratrol Induces DNA Damage-Mediated Cancer Cell Senescence through the DLC1–DYRK1A–EGFR Axis. Food Funct..

[75] Demeyer A., Benhelli-Mokrani H., Chenais B., Weigel P., Fleury F. (2021). Inhibiting Homologous Recombination by Targeting RAD51 Protein. Biochim. Et Biophys. Acta (BBA) Rev. Cancer.

[76] Yang Y., Ke J., Han X., Wuddineh W.A., Song G.Q., Zhong G.Y. (2022). Removal of a 10-Kb *Gret1* Transposon from *VvMybA1* of *Vitis vinifera* cv. Chardonnay. Hortic. Res..

[77] Gharari Z., Bagheri K., Danafar H., Sharafi A. (2020). Enhanced Flavonoid Production in Hairy Root Cultures of Scutellaria Bornmuelleri by Elicitor Induced Over-Expression of MYB7 and FNSП2 Genes. Plant Physiol. Biochem..

[78] Tariq H., Asif S., Andleeb A., Hano C., Abbasi B.H. (2023). Flavonoid Production: Current Trends in Plant Metabolic Engineering and De Novo Microbial Production. Metabolites.

[79] Davuluri G.R., Van Tuinen A., Fraser P.D., Manfredonia A., Newman R., Burgess D., Brummell D.A., King S.R., Palys J., Uhlig J. (2005). Fruit-Specific RNAi-Mediated Suppression of DET1 Enhances Carotenoid and Flavonoid Content in Tomatoes. Nat. Biotechnol..

[80] Zhu Q., Yu S., Zeng D., Liu H., Wang H., Yang Z., Xie X., Shen R., Tan J., Li H. (2017). Development of “Purple Endosperm Rice” by Engineering Anthocyanin Biosynthesis in the Endosperm with a High-Efficiency Transgene Stacking System. Mol. Plant.

[81] Sun B., Zhu Z., Cao P., Chen H., Chen C., Zhou X., Mao Y., Lei J., Jiang Y., Meng W. (2016). Purple Foliage Coloration in Tea (*Camellia sinensis* L.) Arises from Activation of the R2R3-MYB Transcription Factor CsAN1. Sci. Rep..

[82] Wang N., Xu H., Jiang S., Zhang Z., Lu N., Qiu H., Qu C., Wang Y., Wu S., Chen X. (2017). MYB12 and MYB22 Play Essential Roles in Proanthocyanidin and Flavonol Synthesis in Red-Fleshed Apple (*Malus sieversii* f. *Niedzwetzkyana*). Plant J..

[83] Fatihah H.N.N., Moñino López D., van Arkel G., Schaart J.G., Visser R.G.F., Krens F.A. (2019). The ROSEA1 and DELILA Transcription Factors Control Anthocyanin Biosynthesis in Nicotiana Benthamiana and Lilium Flowers. Sci. Hortic..

[84] Schaart J.G., Dubos C., Romero De La Fuente I., van Houwelingen A.M.M.L., de Vos R.C.H., Jonker H.H., Xu W., Routaboul J.-M., Lepiniec L., Bovy A.G. (2013). Identification and Characterization of MYB-BHLH-WD40 Regulatory Complexes Controlling Proanthocyanidin Biosynthesis in Strawberry (Fragaria × Ananassa) Fruits. New Phytol..

[85] Terrier N., Torregrosa L., Ageorges A., Vialet S., Verriès C., Cheynier V., Romieu C. (2009). Ectopic Expression of VvMybPA2 Promotes Proanthocyanidin Biosynthesis in Grapevine and Suggests Additional Targets in the Pathway. Plant Physiol..

[86] Huang Y.-F., Vialet S., Guiraud J.-L., Torregrosa L., Bertrand Y., Cheynier V., This P., Terrier N. (2014). A Negative MYB Regulator of Proanthocyanidin Accumulation, Identified through Expression Quantitative Locus Mapping in the Grape Berry. New Phytol..

[87] Yu Y., Guo D., Li G., Yang Y., Zhang G., Li S., Liang Z. (2019). The Grapevine R2R3-Type MYB Transcription Factor VdMYB1 Positively Regulates Defense Responses by Activating the Stilbene Synthase Gene 2 (VdSTS2). BMC Plant Biol..

[88] Deluc L., Bogs J., Walker A.R., Ferrier T., Decendit A., Merillon J.M., Robinson S.P., Barrieu F. (2008). The Transcription Factor VvMYB5b Contributes to the Regulation of Anthocyanin and Proanthocyanidin Biosynthesis in Developing Grape Berries. Plant Physiol..

[89] Akagi T., Ikegami A., Tsujimoto T., Kobayashi S., Sato A., Kono A., Yonemori K. (2009). DkMyb4 Is a Myb Transcription Factor Involved in Proanthocyanidin Biosynthesis in Persimmon Fruit. Plant Physiol..

[90] Anupa T., Sahijram T., Samarth L. (2016). In Vitro Shoot Induction of Three Grape (*Vitis vinifera* L.) Varieties Using Nodal and Axillary Explants. BioScan.

[91] Fizikova A.Y. (2023). Microclonal Propagation of Elite Industrial Grape Cultivars (*Vitis vinifera* L.). Proc. Appl. Bot. Genet. Breed..

[92] Weigel D., Glazebrook J. (2006). Transformation of Agrobacterium Using the Freeze-Thaw Method. CSH Protoc..

[93] Konagaya K.I., Nanasato Y., Taniguchi T. (2020). A Protocol for Agrobacterium-Mediated Transformation of Japanese Cedar, Sugi (*Cryptomeria japonica* D. Don) Using Embryogenic Tissue Explants. Plant Biotechnol..

[94] Conant D., Hsiau T., Rossi N., Oki J., Maures T., Waite K., Yang J., Joshi S., Kelso R., Holden K. (2022). Inference of CRISPR Edits from Sanger Trace Data. CRISPR J..

[95] Park J., Lim K., Kim J.S., Bae S. (2017). Cas-Analyzer: An Online Tool for Assessing Genome Editing Results Using NGS Data. Bioinformatics.

[96] Kamlesh R.P., Narpat S.S., Graeme P.B., Trevor A.T. (1984). Isolation and Culture of Protoplasts 388 from Cytoledons of Pinus Coulteri D. Don. Plant Cell Tissue Organ Cult..

[97] Nikolaeva T.N., Lapshin P.V., Zagoskina N.V. (2021). Method for Determining the Total Content of Phenolic Compounds in Plant Extracts with Folin-Denis Reagent and Folin-Chocalteu Reagent: Modification and Comparison. Khimiya Rastit. Syr’ya.

[98] Altman D.G., Machi D., Bryan T.N., Gardn M.J. (2000). Statistics with Confidence.

[99] Richardson J.T. (2011). The Analysis of 2 × 2 Contingency Tables—Yet Again. Stat. Med..

[100] Campbell I. (2007). Chi-Squared and Fisher–Irwin Tests of Two-by-Two Tables with Small Sample Recommendations. Stat. Med..

