# Supplementary material for: A New Approach for CRISPR/Cas9 Editing and Selection of Pathogen-Resistant Plant Cells of Wine Grape cv. ‘Merlot’
Source: Int J Mol Sci. 2024 Sep 17;25(18):10011. doi: 10.3390/ijms251810011 (PMC11432302; doi:10.3390/ijms251810011)
Supplement: Supplementary file 1 [file ijms-25-10011-s001.zip › ijms-3174543-supplementary.pdf]

Table S1. Primer list

Primer name and sequence (5′→ 3′)	Purpose
Seq7_156F: ttggatcggtaaggctgctt	*MLO7* sequence verification
MLO7_*R*: atctcatgttgggttcggatt	
P6-VvU6.2_F: cactagggatgactgcctgga	Promoter VvU6.2 sequence amplification
P6-VvU6.2_R: cgcaggggccatgctaatct	
UBQ2-F: gctgacggagtcgttagtca	Promoter VvUBQ2 sequence amplification
UBQ2-R: tctacacagatctaagagag	
121: agatctgtcgatcgaccatggtgagcaagggcgagg	GFP-P2A-HygR fusion PCR
122: gtacagctcgtcgccagtcttgtacagctcgtccatgccg	
123: gggatcactctcggcatggacgagctgtacaagactggcgac	
124: cttctcgacagacgtcgcggtgagttcaggctttttcatggtg	
125: ggggatccaccggtcgccaccatgaaaaagcctgaactcac	
126: agagtcccgcctattcctttgccctcggacg	
127: aaaggaataggcgggactctggggttcg	
128:attaattcgggaagttcctattctctagaaagtataggaacttcgcctgcggaga tctggattttag	
129: taggaacttcccgaattaattcggcgttaattcag	
130: acggagccgattttgaaaccgcgggcggtgatcacaggcagc	
131: gaagtgagctcatattgaaagttaatatgtattatt	HSP-FLP amplification
132: acataatatatcaaattggctcatcaccttcc	
133: atggctcctaagaagaagaggaaggtgatgagccaatttgatatattatg	
134: atgattacaggcctccgatctagtaacatagatgacaccgc	
1F_FRT: ctcttaattaatagggataacaggg	Fusion PCR and cloning
2R_FRT: ccttcagtttgaagttcctattctctagaaag	
3F_preP: taggaacttcaaactgaaggcgggaaac	
4R_preP: aaggtgagatcttcatatcttaagaatgaagatgt	
5F_VvU6– 26: agatatgaagatctcaccttctggtggggaaca	
6R_VvU6– 26: gttggtctcccaatctctaagcgtttgctcctg	
7F_preUBQ: ttagagattgggagaccaacccagtggac	
7F′preUBQSbf/Stu: catggcaggccttcaacatggtggagcacg	
8R′preUBQSbf/Stu: tgaaggcctgccatgcaagcttattggtttatctc	
8R_preUBQ: ctccgtcagcattgccctttggtcttctgag	
9F_VvUBQP: aaagggcaatgctgacggagtcgttagtc	
10R_VvUBQ: tgaggtcgagtctacacagatctaagagagtacc	
11F_Cas9: tctgtgtagactcgacctcaacacaacatatac	
12R_Cas9SbfI: ctcctgcaggtagcagatgcg	
prF Hyper/m1NcoI: gatcacccttgggccaatagagaatgcgt	HyPer sequence PCR mutagenesis
PrR Hyper/m1NcoI: ggcccaagggtgatcttcatagattgctaaa	

prF Hyper/m2NcoI: cctgtcccttggccaacattagtaacaacctt	
prR Hyper/m2NcoI: tggccaagggacaggaagttttccagttgt	
prF_HyperNcoI: gccaccatggaaatggcttctcaacagg	
prR_HyperSacII: gaaaccgcggccgcgggcggtgatcacaggc	
prRHyperfusion: gagtcccgcttacactgcttgcttcaaaacc	
prFHyperfusion: gaagcaagcagtgtaagcgggactctggggttc	Hyper sequence fusion PCR and cloning
gRNA 7.7F: attgttggttcttcccaaagcca	
gRNA 7.7R: aaactggctttgggaagaaccaa	gRNA expression
Seq_1_F: ttctcttaggtttacccgccaa	
Seq_1_R: tcttgaaggttcgatgcctaatca	
Seq_2_F: cttgaaccgtagctttcgttttct	
Seq_2_R: aactttgttttagggcgactgc	
Seq_3_F: ttggggtacagtctatgcctcg	
Seq_3_R: cctgttcaggaaccggatcaaa	
Seq_4_F: cacgatcgacattgatctggct	
Seq_4_R: acggactagccttattttaacttgc	
Seq_5_F: gctcacagccaaactatcaggt	
Seq_5_R: cacggcatctagatgtcccttg	
Seq_10_F: aggatcttcttagaaagcagagaacc	
Seq_10_R: acgtacttaaccttggtaagctcg	
Seq_11_F: catcgagagaatgaccaacttcga	
Seq_11_R: tcgtggataagctgcatgaagt	
Seq_12_F: cggaatcagagataagcagtctgg	
Seq_12_R: atcagaacgggtaagaaccttgt	
Seq_13_F: acgttgatcaggagcttgacat	
Seq_13_R: tttccgatctcctgctcagact	
Seq_14_F: tgttggaaccgctcttatcaaga	
Seq_14_R: tggcaacttgatgataagatccttct	
Seq_15_F: caccatcatggagcgttcttct	
Seq_15_R: tcctccaagctgagaaagatcg	
Seq_16_F: gctccagctgctttcaagtact	
Seq_16_R: ttcaaacaaaacactgaaaccatagag	
Seq_18_F: tatccccttcgtcctcgtcaaa	
Seq_18_R: gatctcccagatgctttcaccc	
Seq_19_F: tcgaatcatcggaagaagcaga	
Seq_19_R: ttagtcaactccgttaggccct	
Seq_20_F: tgcgccttatccaatctttgct	
Seq_20_R: tgccaaatgtttgaacgatcgc	Vector sequencing

Seq_21_F: tcagttccagcgacttgattcg	
Seq_21_R: tgggagatgaggaggctttctt	
Seq_22_F: ccactaattcgtcctgctggtt	
Seq_22_R: gcaacgcaattaatgtgagttagc	
Seq_23_F: atccgctcacaattccacacaa	
Seq_23_R: tcaatagccctttggtcttctga	
Seq_24_F: acgttccaaccacgtcttcaaa	
Seq_24_R: tgaacttgtggccgtttacgtc	
Seq_25_F: agctttcgcagatctgtcgatc	
Seq_25_R: atgggggtgttctgctggta	
Seq_29_F: gggttcctatagggtttcgctc	
Seq_29_R: actgttccttgcattctaaaacctt	
Seq_30_F: tcggctccgtcgatactatgtt	
Seq_30_R: aattcggctaagcggctgtcta	
Seq_31_F: ttatcgagctgtatgcggagtg	
Seq_31_R: cagcagattacgcgcagaaaaa	
Seq_32_F: acgtgagttttcgttccactga	
Seq_32_R: ctcaagtcagaggtggcgaaac	
Seq_33_F: gtgagctatgagaaagcgccac	
Seq_33_R: agacggtcacagcttgtctg	
Seq_34_F: tatcgctacgtgactgggtcat	
Seq_34_R: ctagcaggggaaaaaggtcgaa	
Seq_35_F: gtacggctttgggttcccaat	
Seq_35_R: gtacgaagaaggccaagaacgg	
Seq_36_F: tcagctagctcgatctcgatgt	
Seq_36_R: ccgctgatcgaatccgcaaa	
1F_FRTslic: cctgtcaaacactgatagtttctcttaattaatagggataaca	
2R_FRT: ccttcagtttgaagttcctattctctagaaag	
3F_preP: taggaacttcaaactgaaggcgggaaac	
4R_preP: aaggtgagatcttcatatcttaagaatgaagatgt	
5F_VvU6-26: agatatgaagatctcaccttctggtggggaaca	
6R_VvU6-26: gttggtctcccaatctctaagcgtttgctcctg	
7F_preUBQ: ttagagattgggagaccaacccagtggac	
7F'preUBQSbf/Stu: catggcaggccttcaacatggtggagcacg	
8R'preUBQSbf/Stu: tgaaggcctgccatgcaagcttattggtttatctc	
8R_preUBQ: ctccgtcagcattgccctttggtcttctgag	
9F_VvUBQP: aaagggcaatgctgacggagtcgttagtc	
10R_VvUBQ: tgaggtcgagtctacacagatctaagagagtacc	AtU6-26, 35S promoters replacement by VvU6-26, VvUBQ2 promoters

11F_Cas9: tctgtgtagactcgacctcaacacaacatatac	
12R: gccatctcgttggagaaaatctcctgcaggtagcagatgc	
